# Antiseptic 9-Meric Peptide with Potency against Carbapenem-Resistant *Acinetobacter baumannii* Infection

**DOI:** 10.3390/ijms222212520

**Published:** 2021-11-20

**Authors:** Manigandan Krishnan, Joonhyeok Choi, Ahjin Jang, Young Kyung Yoon, Yangmee Kim

**Affiliations:** 1 Department of Bioscience and Biotechnology, Konkuk University, Seoul 05029, Korea; biomani1@konkuk.ac.kr (M.K.); jun9688@konkuk.ac.kr (J.C.); ajin931017@konkuk.ac.kr (A.J.); 2 Department of Internal Medicine, Division of Infectious Diseases, College of Medicine, Korea University Anam Hospital, Korea University, Seoul 02841, Korea; young7912@korea.ac.kr

**Keywords:** antimicrobial peptide, *A. baumannii*, carbapenem-resistance, sepsis

## Abstract

Carbapenem-resistant *A. baumannii* (CRAB) infection can cause acute host reactions that lead to high-fatality sepsis, making it important to develop new therapeutic options. Previously, we developed a short 9-meric peptide, Pro9-3D, with significant antibacterial and cytotoxic effects. In this study, we attempted to produce safer peptide antibiotics against CRAB by reversing the parent sequence to generate R-Pro9-3 and R-Pro9-3D. Among the tested peptides, R-Pro9-3D had the most rapid and effective antibacterial activity against Gram-negative bacteria, particularly clinical CRAB isolates. Analyses of antimicrobial mechanisms based on lipopolysaccharide (LPS)-neutralization, LPS binding, and membrane depolarization, as well as SEM ultrastructural investigations, revealed that R-Pro9-3D binds strongly to LPS and impairs the membrane integrity of CRAB by effectively permeabilizing its outer membrane. R-Pro9-3D was also less cytotoxic and had better proteolytic stability than Pro9-3D and killed biofilm forming CRAB. As an LPS-neutralizing peptide, R-Pro9-3D effectively reduced LPS-induced pro-inflammatory cytokine levels in RAW 264.7 cells. The antiseptic abilities of R-Pro9-3D were also investigated using a mouse model of CRAB-induced sepsis, which revealed that R-Pro9-3D reduced multiple organ damage and attenuated systemic infection by acting as an antibacterial and immunosuppressive agent. Thus, R-Pro9-3D displays potential as a novel antiseptic peptide for treating Gram-negative CRAB infections.

## 1. Introduction

Gram-negative sepsis is caused by an unregulated immune response to infection in which immune cells are activated by lipopolysaccharide (LPS) produced from the bacterial outer membrane, resulting in severe inflammation, organ failure, and even death [1,2]. The term “ESKAPE” comprises six highly antibiotic-resistant pathogens: *Enterococcus faecium*, *Staphylococcus aureus*, *Klebsiella pneumoniae*, *Acinetobacter baumannii*, *Pseudomonas aeruginosa*, and *Enterobacter species*, which account for the majority of bacteremia cases and surgical-site infections in healthcare settings [3]. Among these ESKAPE pathogens, Gram-negative *A. baumannii* has been identified as a significant opportunistic pathogen that causes lethal sepsis with a high death rate in hospitals [4]. It infects roughly 1 million individuals each year, and 44% of clinical isolates are multidrug-resistant (MDR) *A. baumannii* [5,6]. *A. baumannii*, as an opportunistic pathogen, can also cause coinfection, particularly when combined with viral respiratory tract infections in hospitalized patients, and secondary infection in COVID-19 patients has recently been widely reported [7]. Some of the most common mechanisms of resistance in these isolates include penicillin-binding protein mutations, porin loss, antibiotic target site mutations, and efflux pump overexpression [8].

Current first-line treatments for *A. baumannii* include β-lactams or carbapenem antibiotics, such as imipenem and meropenem; however, the emergence of carbapenem-resistant *A. baumannii* (CRAB) strains with β-lactamase hydrolysis and carbapenemase overproduction has restricted therapeutic options [9]. Worryingly, the mechanism of resistance to colistin and/or tigecycline in CRAB strains has been linked to structural alterations in LPS via mutations in the genes coding for lipoxygenase and polymyxin-resistance-associated response regulator (pmr)-A/B [10,11]. Antibiotic resistance is associated with biofilm formation, in which secreted substances such as extracellular matrix polysaccharides, proteins, and DNA adhere to biotic or abiotic surfaces, increasing the virulence and antibiotic resistance of CRAB isolates in immunocompromised patients [12,13]. Carbapenems such as doripenem, imipenem and meropenem are generally considered as a last-line treatment for multi-drug resistant *A. baumannii*. Among many carbapenem-hydrolyzing oxacillinase-encoding genes, OXA-23 is widespread in Korea and the number of antibiotics available to treat CRAB-induced sepsis are decreasing [14]. Since developing therapeutic agents to treat sepsis caused by CRAB is highly difficult, there is an immediate and urgent need for the development of effective antiseptic compounds.

Antimicrobial peptides (AMPs) are part of the first line of defense in all living host species and have emerged as a new class of antibiotics for treating infections [15]. Many AMPs have a broad spectrum of bactericidal activity; limited resistance potential; immunomodulatory capabilities; and anti-inflammation, and antibiofilm effects that are uncommon for standard antibiotics [16,17,18]. Some of the AMP that are effective against Gram-negative infections include human lactoferrin-derived hLF1-11 which is in phase 1 trials [19]. P-113, which is derived from human histatin-5, and Omiganan, which is derived from indolicidin, are both in phase 3 trials [20,21]. Antiviral properties of AMPs have been demonstrated and Hp1090 peptide from the scorpion *Heterometrus petersii* was the first natural antiviral peptide exhibiting viricidal activity against the hepatitis C virus [22,23]. Recently, a natural lectin-like human defensins-5 (HD5) peptide secreted by Paneth cells in the Lieberkühn crypts competitively blocked SARS-CoV-2 ACE2 receptors and prevented viral recognition and attachment [24]. In the context of *Acinetobacter* species, numerous AMPs have been studied, for example, the cyclic peptide cathelicidin-BF15-a4 (ZY4) was found to effectively fight MDR *A. baumannii* infections in a mouse model of septicemia [25]. In addition, the cecropin peptide Cec4 was shown to exert significant anti-CRAB effects by reducing biofilm development [26]. However, due to their cytotoxicity, limited half-life, proteolytic breakdown, and production problems, only a few AMPs have been employed in clinical practice [12,27].

To counteract the sensitivity of AMPs to proteolysis, many peptidomimetics have been developed that include D-amino acids [28], unnatural amino acids [29], peptide backbone modifications [30], cyclization [31], Triazole-alterations [32], and secondary structure-inducing templates [33]. However, in the vast majority of cases, partial or total peptide modification reduces or even inhibits biological activity [34]. Another intriguing method for producing peptidomimetics is the retro inversion (RI) strategy, which uses D-amino acids as stable surrogates for L-amino acids that are delivered in a reverse (retro) order to the original molecule. Due to their inverted chirality, these peptides are less susceptible to proteolytic digestion, resulting in a longer half-life [35,36]. Indeed, some of the RI analogues of AMPs in clinical trials are currently displaying good specificity against ESKAPE pathogens, such as RI-omiganan [37], RI-CAMEL [38], and RI-temporin A [39].

Protaetiamycine is an insect defensin that has strong antibacterial activities against both Gram-negative and Gram-positive bacteria but has significant cytotoxicity [40,41]. Previously, we designed two 9-meric peptides, namely Pro9-3 and Pro9-3D (enantiomeric peptide), based on the structure–activity relationships of this defensin. To reduce cytotoxicity against mammalian cells, we attempted to design 10-meric peptides (Pro10-1 and its enantiomer, Pro10-1D) by adding an Arg residue at the N-terminus of Pro9-3, which increased bacterial cell selectivity [42]. In this study, we aimed to develop potent short 9-meric peptides with high selectivity and low cytotoxicity for treating CRAB-induced sepsis. We designed R-Pro9-3 and R-Pro9-3D using the RI strategy and tested their antibacterial activities against clinical CRAB isolates using in vitro and in vivo models of sepsis. Here, we describe the development of a short protease-resistant, antibiofilm, antiseptic peptide antibiotic to treat CRAB infection.

## 2. Results

### 2.1. Peptide Design

**Figure 1:**
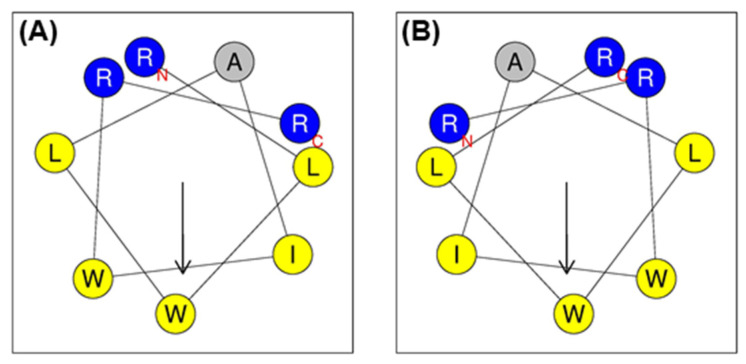
Helical wheel projections of (**A**) Pro9-3 and (**B**) R-Pro9-3 using HeliQuest program (http://heliquest.ipmc.cnrs.fr/cgi-bin/ComputParams.py), Date accessed: 12 September 2021. The positively charged amino acid residues are shown in blue and hydrophobic residues are shown in yellow, except for alanine, which is represented by the color gray. Arrows (helical hydrophobic moment), N (N-terminal region), and C (C-terminal region).

Previously, we designed Pro9-3 from the active site of the insect defensin, protaetiamycine (43 amino acids); however, despite having strong antibacterial activity, Pro9-3 and its enantiomeric peptide (Pro9-3D) showed severe cytotoxicity against mammalian cells (Table 1). By adding one more Arg to the N-terminus of these peptides, we produced Pro10-1 and Pro10-1D with two sequential Arg residues at their N-termini, which showed greater bacterial cell selectivity than their parent 9-mer peptides [42]. Here, we retained the short length of nine amino acids to ensure the same cationicity, hydrophobicity, and antibacterial activity. However, to decrease the cytotoxicity of Pro9-3, we reversed the peptide sequence, resulting in a retro peptide (R-Pro9-3) with two sequential Arg residues at the N-terminus (Table 1). In addition, we designed a retro-D-peptide (R-Pro9-3D) by replacing the L-amino acid in R-Pro9-3 with a D-amino acid. The helical wheel projections of all peptides exhibited amphipathicity (Figure 1), implying that these peptides may form amphipathic α-helical structures in the bacterial membrane and effectively permeabilize the bacterial membrane.

### 2.2. Antibacterial Activity of Peptides against Carbapenem-Resistant A. baumannii (CRAB)

The antimicrobial activities of R-Pro9-3, R-Pro9-3D, their parent peptides (Pro9-3 and Pro9-3D), and controls (imipenem, meropenem, and melittin) were examined against standard Gram-negative bacteria and clinical isolates of carbapenem-resistant *E. coli* (CREC), *A. baumannii* (CRAB), and *K. pneumoniae* (CRKP). As shown in Table 2, R-Pro9-3D showed superior antibacterial activities against Gram-negative strains compared to its parent enantiomer (Pro9-3D), whereas R-Pro9-3 exerted weak bactericidal effects similar to its native form (Pro9-3) when compared with the melittin control and conventional antibiotics. We next evaluated the effects of these four peptides on carbapenem-resistant strains and found that R-Pro9-3D displayed the strongest activity, especially against CRAB (Table 2), with the following order of geometric mean (GM) values: R-Pro9-3D (6.3) < Pro9-3D (8.0) < melittin (13.1) < Pro9-3 (25.6) < R-Pro9-3 (27.4). In addition, R-Pro9-3D had the highest therapeutic index value (31.7), followed by Pro9-3D (25.0) and Pro9-3 (7.8), which had a similar value to R-Pro9-3 (7.3). Conversely, melittin (0.2) had a very low therapeutic index value. Since all the CREC, CRAB, and CRKP strains had already acquired resistance to carbapenem antibiotics, such as imipenem and meropenem, these antibiotics showed elevated MIC values against the carbapenem-resistant strains. 

**Figure 2:**
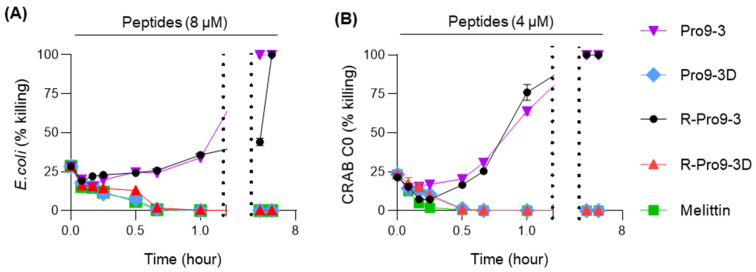
Time-killing curves of peptides against (**A**) *E. coli* and (**B**) CRAB C0 strains. For each peptide, the bacterial cells were challenged with the R-Pro9-3D MIC (8 μM for *E. coli* and 4 μM for CRAB C0) and the killing abilities were monitored for different time intervals (0–4 h). The values are expressed as the mean ± SEM of three independent experiments.

Notably, Pro9-3D and R-Pro9-3D exhibited significant bactericidal effects against *E. coli* and CRAB C0 (Table 2); therefore, we further examined the potency of these peptides using time-killing assays. As shown in Figure 2, R-Pro9-3D and Pro9-3D effectively killed *E. coli* within 4 h at 8 μM and killed CRAB C0 within 2 h at 4 μM. However, the L-form peptides, Pro9-3 and R-Pro9-3, were unable to kill *E. coli* and CRAB C0 within 4 h, suggesting that Pro9-3D and R-Pro9-3D can kill these bacteria more effectively and rapidly than their parent peptides.

### 2.3. Mechanism of Antibacterial Activity against CRAB

**Figure 3:**
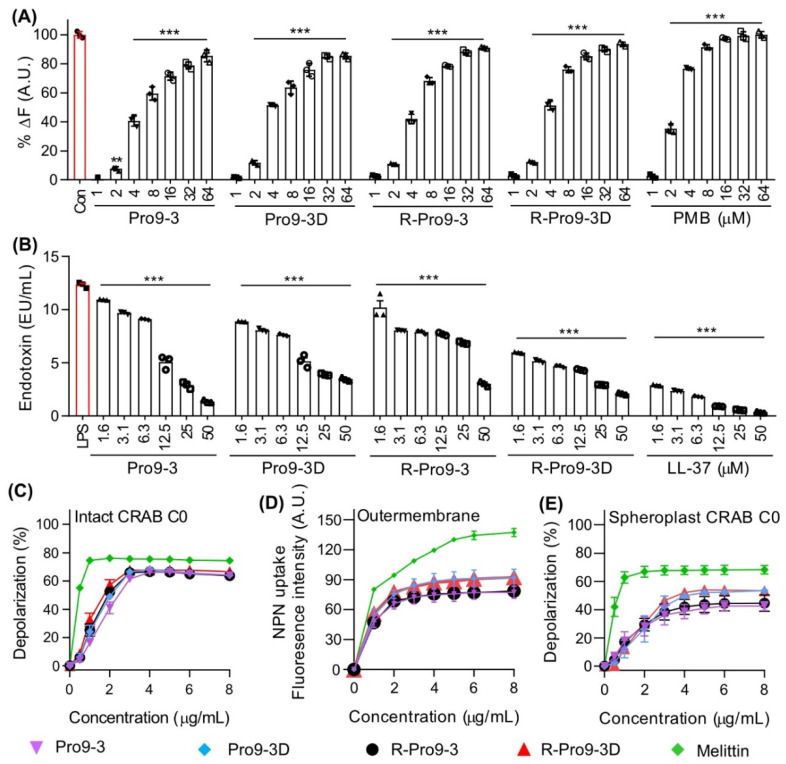
Antibacterial mechanism of the peptides. (**A**) Lipopolysaccharide (LPS) binding affinities peptides based on displacement assays with BODIPY-TR-cadaverine fluorescent dye. (**B**) Limulus amebocyte lysate (LAL) assay showing the LPS neutralization capacities of peptides and LL-37 control. (**C**) The concentration-dependent depolarization of intact CRAB C0, (**D**) Outer membrane permeability assessed by NPN uptakes induced by peptides. (**E**) Cytoplasmic membrane depolarization capacities by peptides on LPS-layer removed spheroplast CRAB C0 cells. Melittin was used as control. Statistical analysis was performed using two-way analysis of variance (ANOVA) with Dunnett’s comparison test. The values are expressed as the mean ± SEM of three independent experiments and are statistically significant at *** *p* < 0.001; ns, not significant.

Next, we investigated the antimicrobial mechanism of the different peptides examined in this study. LPS is the main component of the outer membrane of Gram-negative bacteria and induces TLR4-mediated inflammatory signaling; therefore, peptides with good anti-endotoxin activities that can clear bacterial LPS have attracted increasing attention for treating Gram-negative infections. As such, we investigated the LPS binding affinity of Pro9-3 and its analogs using BODIPY-TR-cadaverine (BC) displacement assays (Figure 3A). All peptides showed excellent LPS-binding capacities, with Pro9-3, Pro9-3D, R-Pro9-3, and R-Pro9-3D (4 μM) increasing BC displacement by 42.0%, 51.2%, 40.6%, and 51.3%, respectively, compared to the well-known LPS-neutralizing peptide PMB (76.6%). In addition, we evaluated the LPS-neutralizing capacity of the peptides using limulus amebocyte lysate (LAL) assays. As shown in Figure 3B, all peptides neutralized LPS in a concentration-dependent manner compared to the control, LL-37 (1.6 μM), which is a well-known LPS-neutralizing peptide (R-Pro9-3D, 51.6%; Pro9-3D, 27.9%; Pro9-3, 11.3%; R-Pro9-3, 17.3%; and LL-37, 76.5%). Thus, our findings suggest that R-Pro9-3D may possess greater LPS-recognition capabilities than its parent peptides.

To further understand the antibacterial mechanism of the peptides against CRAB, we examined their ability to depolarize its outer membrane. First, we investigated the depolarization of intact CRAB by each peptide, as indicated by an increase in the intracellular distribution of the diSC_3_-5 fluorophore. As shown in Figure 3C, all peptides increased diSC_3_-5 fluorescence in a concentration-dependent manner in a similar range to that for melittin. In particular, 4 μM Pro9-3, Pro9-3D, R-Pro9-3, R-Pro9-3D, and melittin increased depolarization by 66.3, 68.4, 66.7, 67.8, and 75.6%, respectively, suggesting that these peptides target the CRAB membrane. Since a major component of the outer membrane of CRAB is LPS, which our peptides bound to and neutralized effectively, we compared the abilities of each peptide to depolarize the outer membrane of CRAB using 1-N-phenylnapthylamine (NPN) uptake. NPN exhibits strong fluorescence in the hydrophobic interior of a lipid bilayer; therefore, outer membrane permeabilization increases fluorescence. R-Pro9-3D and Pro9-3D induced greater NPN uptake than their L-form peptides, indicating that they may interact with LPS in the outer membrane of CRAB more effectively than the L-form peptides (Figure 3D). We also prepared CRAB spheroplasts by removing LPS and peptidoglycan from the CRAB outer membrane. As shown in Figure 3E, 4 μM Pro9-3, Pro9-3D, R-Pro9-3, R-Pro9-3D, and melittin increased diSC_3_-5 fluorescence by 38.7%, 49.8%, 42.0%, 52.2%, and 67.9%, respectively. Notably, the CRAB spheroplasts were depolarized by 22% less than were intact CRAB cells. Taken together, these results suggest that the antibacterial mechanism of these peptides involves a strong interaction with LPS in the outer membrane and permeabilization of the CRAB membrane.

### 2.4. Effect of Peptides on Killing Biofilm Forming Bacteria

**Figure 4:**
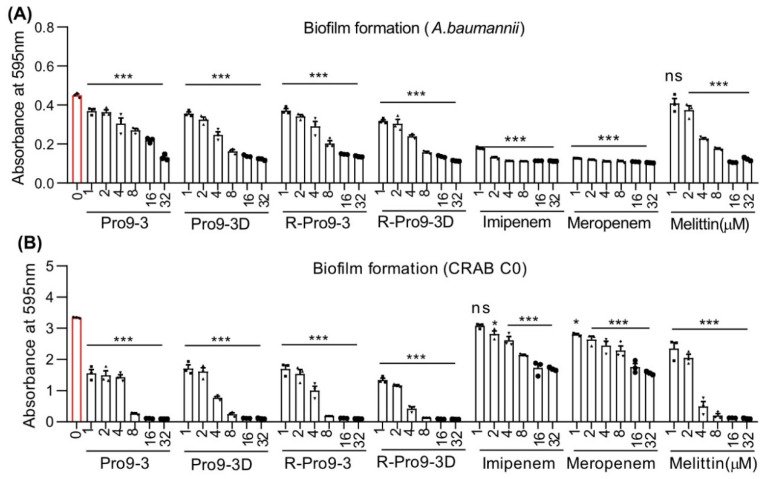
Pro9-3 and its analogs have biofilm inhibition properties against CRAB. The antibiofilm activities of peptides (0–32 μM, 16 h) on (**A**) *A. baumannii* and (**B**) CRAB C0 strains were measured by crystal violet staining and the number of attached cells were read at 595 nm. Melittin, meropenem and imipenem were used as a control. Data are shown as the mean ± SEM (*n* = 3) and are statistically significant at * *p* < 0.05; *** *p* < 0.001; ns, not significant (two-way analysis of variance (ANOVA) with Dunnett’s comparison test).

Infections with *A. baumannii* are more common in hospitalized patients and are frequently multidrug resistant. It has the ability to form biofilms, which appear to function as a matrix-enclosed barrier in harsh environments, and it also reduces antibiotic penetration [43,44]. In hospital settings, the effect of antibiotic resistance levels on bio-film formation in carbapenem-resistant Gram-negative bacteria is a serious health-care issue [45]. Hospital effluent water, living tissues such as skin, mucosa, and wounds, and medical equipment such as urinary catheters and other ventilator materials are the primary sources of biofilm formation [46,47,48]. Thus, we next investigated the biofilm inhibiting capacities of R-Pro9-3D on *A. baumannii* and CRAB C0 strains using crystal violet staining. Treatment with all Pro9-3 peptides, including melittin, and control antibiotics killed biofilm-forming *A. baumannii* for 16 h (Figure 4A). Notably, the peptide R-Pro9-3D are superior in killing biofilm-forming CRAB C0 (Figure 4B), as evidenced by significantly reduced crystal-violet absorbance (0.4 at OD_595_) at their MIC when compared to other peptides, whereas imipenem and meropenem were unable to inhibit due to acquired resistance to these antibiotics. These findings imply that R-Pro9-3D can be effective in eradicating preformed biofilms and/or translocate the biofilm matrix due to their strong membrane-permeable nature thereby causes CRAB C0 biofilm inhibition.

### 2.5. Resistance of Peptides against Protease Digestion

Proteolytic degradation of peptides in serum limits their application in clinical use due to their reduced bioavailability and half-life span [49]. Thus, we next examined the stability of all peptides were pre-exposed with trypsin and chymotrypsin proteases and the relative growth inhibition of *E. coli*, *A. baumannii* and CRAB C0 was measured (Table 3). The result revealed that Pro9-3 and R-Pro9-3 do not affect the survival of these bacterial strains even at 64 μM, whereas their enantiomeric peptides, Pro9-3D and R-Pro9-3D effectively maintained their bactericidal effects without affecting their MICs, suggesting their greater proteolytic stability by D-amino acid substitution.

### 2.6. Circular Dichroism (CD) Spectroscopy of Peptides 

**Figure 5:**
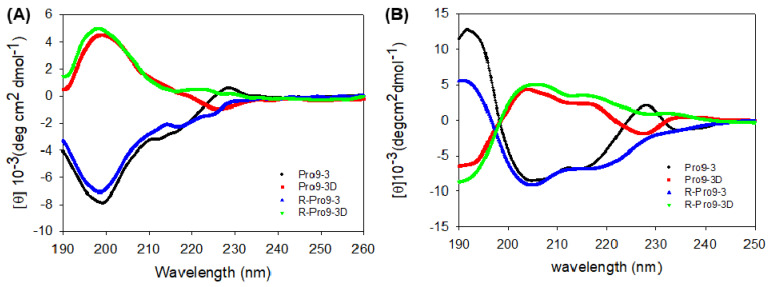
Circular dichroism spectra of Pro9−3, Pro9−3D and their retro-peptides at 100 µM in (**A**) aqueous solution and (**B**) 50 mM dodecylphosphocholine (DPC) micelles acquired with 10 scans: Double negative maxima at 205 and 220 nm reflects the characteristic of α-helical structures.

The secondary structural changes of all the peptides in aqueous solution and membrane-mimicking environment were examined by CD measurements (Figure 5). Our result revealed that all peptides exhibited random coil structures in aqueous environment (Figure 5A) but induced in to fully α-helical structures in the presence of dodecylphosphocholine (DPC) micelles. Notably, the CD spectra of all the peptides in DPC micelle clearly showed double negative maxima or minima at 205 nm and 220 nm, which are typical characteristics of α-helices. Pro9-3D have the opposite shape as L-peptides, which is a common occurrence for distinct amino acid conformations. Since Pro9-3D and its reverse sequence (R-Pro9-3D) exhibiting same chirality, their CD spectra represents the mirror image of the parent peptide. As shown in Figure 5B, R-Pro9-3D showed a slightly higher contents of α-helical structure compared to Pro9-3D in a membrane mimetic environment.

### 2.7. Cytotoxicity of Peptides against Mammalian Cells

**Figure 6:**
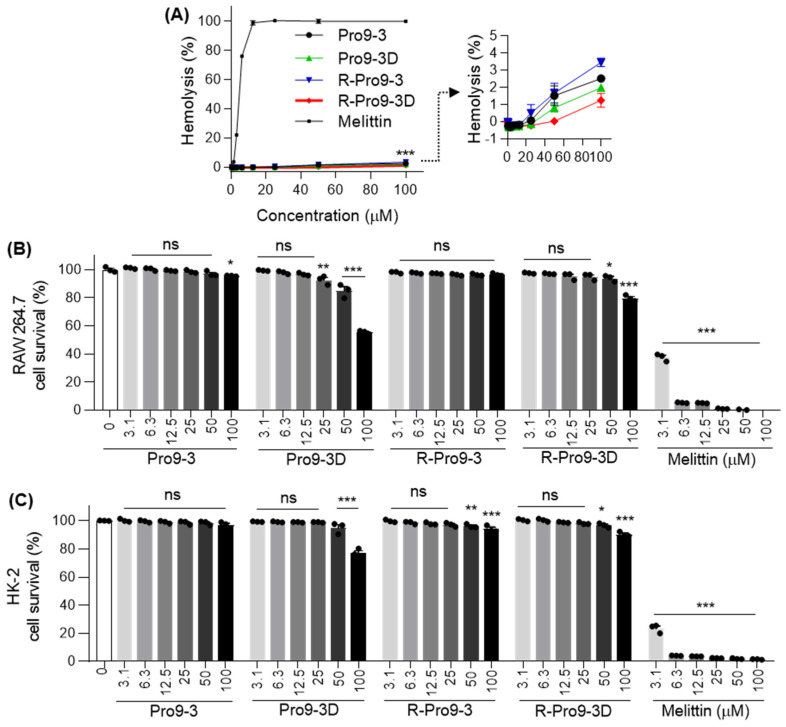
In vitro cytotoxicity of Pro9-3 and their analogues. (**A**) Dose-response curve represent the hemolytic activity of peptides against sheep red blood cell (sRBC). Concentration-dependent (0–100 μM) toxic effects induced by peptides on (**B**) RAW 264.7 murine macrophage cells and (**C**) human kidney (HK)-2 cells for 24 h. Melittin was used as a control. Statistical analysis was performed using two-way analysis of variance (ANOVA) with Dunnett’s comparison test. Values are expressed as mean ± SEM of three independent experiments. * *p* < 0.05, ** *p* < 0.01, *** *p* < 0.001, and ns, non-significant compared with the control.

Next, we investigated the toxic effects of R-Pro9-3 and R-Pro9-3D against different mammalian cells in comparison with their parent peptides (Figure 6). In sheep red blood cells (sRBCs), R-Pro9-3, R-Pro9-3D, Pro9-3 and Pro9-3D induced significantly less hemolytic activity (3.4% and 1.2% vs. 2.5% and 1.9%, respectively) even at 100 μM, whereas melittin, a well-known AMP with broad spectrum antibacterial activity and high cytotoxicity, achieved 100% hemolysis at 25 μM (Figure 6A). As shown in Table 2, R-Pro9-3D had the highest relative selective index (31.7) against the tested Gram-negative bacteria, followed by Pro9-3D, Pro9-3, and R-Pro9-3 (25.0, 7.8, and 7.3, respectively). Further cell-based cytotoxicity analysis revealed that neither R-Pro9-3 nor Pro9-3 (parent) showed toxicity, even at concentrations up to 100 μM, compared to melittin (Figure 6B,C). Conversely, 100 μM Pro9-3D exhibited 44.5% and 22.9% toxicity in RAW 264.7 and human kidney (HK)-2 cells, respectively, and R-Pro9-3D showed one-fold lower toxicity at 20.3 and 9.6%. These results indicate that R-Pro9-3D exhibits greater bacterial cell selectivity and less cytotoxicity than the other peptides.

### 2.8. R-Pro9-3D Suppresses Inflammatory Cytokine Production in Lipopolysaccharide (LPS)-Stimulated RAW 264.7 Cells

**Figure 7:**
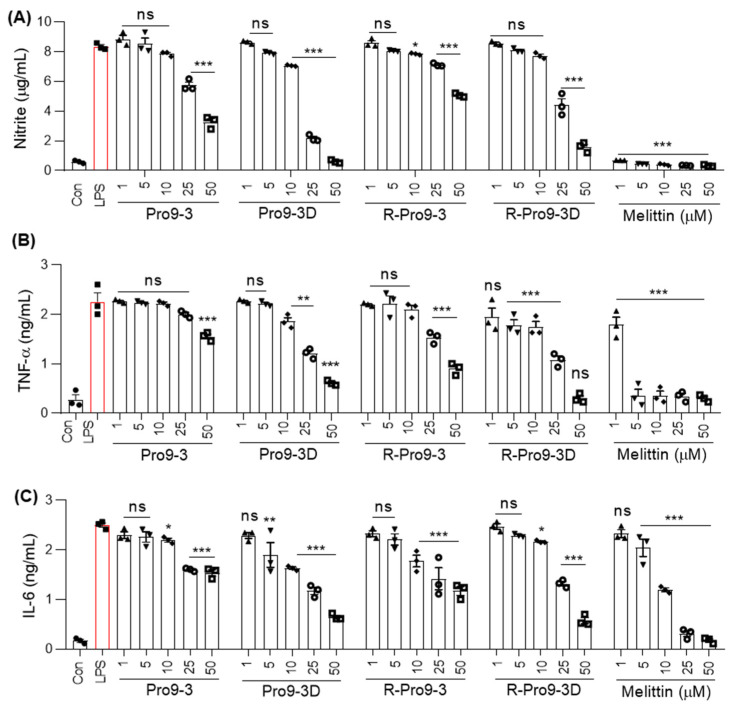
R-Pro19-3D regulates LPS-induced inflammatory cytokine release in vitro. Dose-dependent inhibitory effects by peptides on (**A**) nitrite, (**B**) TNF-α, and (**C**) IL-6 levels in LPS-challenged RAW 264.7 cells. Cells were treated with peptides (0–50 µM) for 1 h followed by LPS stimulation (20 ng/mL) for 16 h. Statistics was performed using two-way analysis of variance with Dunnett’s test. Values are expressed as mean ± SEM (3 independent experiments). Comparisons * *p* < 0.05, ** *p* < 0.01, *** *p* < 0.001, and ns, non-significant compared with LPS control.

Nitric oxide (NO) overproduction is known to be associated with acute and/or chronic inflammation in response to bacterial LPS [50]. Since R-Pro9-3D interacted effectively with LPS and its retro inverted nature preserved its antibacterial activity, we hypothesized that it might regulate LPS-induced inflammatory signaling. Therefore, we examined the inhibitory effects of each peptide against NO production in LPS-stimulated RAW 264.7 cells. As shown in Figure 7A, all peptides significantly inhibited NO secretion in a dose-dependent manner compared to melittin. Notably, R-Pro9-3D exerted the greatest inhibitory effects (47% and 81% at 25 μM and 50 μM, respectively) compared to R-Pro9-3 and their parent peptides. ELISA-based analyses of tumor necrosis factor (TNF)-α and interleukin (IL)-6 levels (Figure 7B,C) revealed significantly elevated cytokine release in LPS-RAW 264.7 cells. Treatment with 25 μM R-Pro9-3D inhibited TNF-α and IL-6 by 52.1% and 47.2%, respectively, while Pro9-3, Pro9-3D, and R-Pro9-3 inhibited TNF-α and IL-6 to a lesser degree. Compared to the other peptides, R-Pro9-3D had greater antibacterial, LPS-binding, antibiofilm, and proteolytic resistance, as well as strong anti-inflammatory effects. Consequently, we selected R-Pro9-3D as a potent candidate antibiotic and analyzed its antiseptic activity in a mouse model of CRAB C0-induced sepsis.

### 2.9. R-Pro9-3D Effectively Damaged the Outer Membrane of CRAB C0 

**Figure 8:**
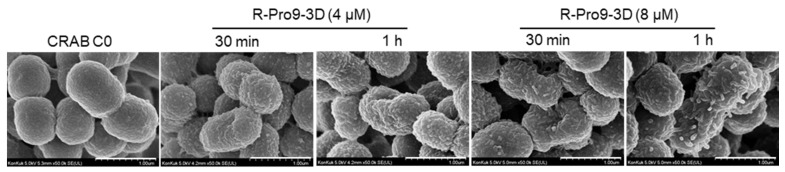
Scanning electron micrographs of CRAB C0 treated with R-Pro9-3D. Control CRAB C0 without peptide treatment. CRAB C0 treated with 4 μM and 8 μM of R-Pro9-3D for 30 min and 1 h at 37 °C. Peptide treatment displays the blisters protruding from the CRAB C0 cells signifies peptide-membrane interaction (Scale bar, 1 μm).

To confirm the antibacterial mechanism, the membrane disruption of CRAB C0, we utilized FE-SEM. As a result of time killing assay in Figure 2, R-Pro9-3D completely killed the bacteria within 1 h of exposure. Therefore, in order to check the membrane change of CRAB C0, it was incubated with R-Pro9-3D at 1 × MIC (4 μM) and 2 × MIC (8 μM) concentrations for 30 min and 1 h, followed by fixation and measurement (Figure 8). As a result, CRAB C0 in its normal state has a smooth surface and intact, but when incubated with R-Pro9-3D at 1 × MIC, the surface becomes rough and caused severe changes of the morphology. As a result of peptide-membrane interaction, R-Pro9-3D treatment at 2 × MIC resulted in significant bubbles protruding from the membrane, supporting the antibacterial mechanism of R-Pro9-3D via the membrane disruption of CRAB C0.

### 2.10. R-Pro9-3D Protects Mice against CRAB-Induced Septic Shock

**Figure 9:**
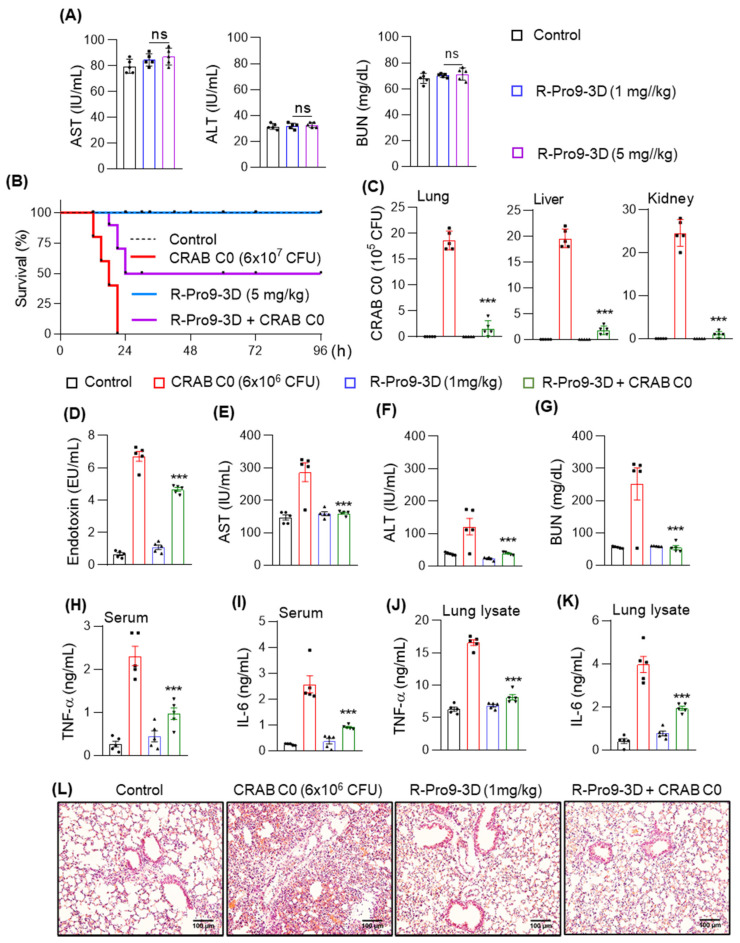
R-Pro9-3D exhibited antiseptic effects in CRAB C0-induced sepsis mouse model. (**A**) R-Pro9-3D did not alter the concentrations of aspartate aminotransferase (AST), alanine aminotransferase (ALT) and blood urea nitrogen (BUN) in response to 1 and 5 mg/kg of peptide treatment. Control mice received phosphate-buffered saline only and the treatment was performed for 24 h. (**B**) R-Pro9-3D prolonged the survival rate of mice infected with CRAB C0. ICR mice (10 per group) were intraperitoneally injected with R-Pro9-3D (5 mg/kg) for 1 h followed by CRAB C0 infection (6 × 10^7^ CFU/mouse) and survival effects were observed for 96 h. In sepsis model, mice (5 mice per group) were treated with R-Pro9-3D (1 mg/kg) for 1 h and the antiseptic analyses were evaluated after CRAB C0 (6 × 10^6^ CFU/mouse, 16 h) infection. (**C**) R-Pro9-3D restricted the bacterial entry in the lung, liver, and kidneys. The corresponding organ lysates were plated onto agar plates and the total no of bacterial colonies were scored. (**D**) R-Pro9-3D effectively normalized the endotoxin levels in CRAB C0-infected mice. (**E**–**G**) Effect of R-Pro9-3D on serum levels of AST, ALT and BUN. (**H**–**K**) R-Pro9-3D inhibited the production of inflammatory cytokines (TNF-α and IL-6) in the serum and lung lysates of CRAB C0-infected mice, as measured by enzyme-linked immunosorbent assay (ELISA). (**L**) Photomicrograph show the modulatory effect of R-Pro9-3D on neutrophil infiltration in the hematoxylin and eosin (H&E)-stained lung tissues (Magnification 20×, Scale bar 100 μm). Statistics was performed using (**A**,**C**–**K**) one-way analysis of variance followed by Dunnett’s test and (**B**) Kaplan–Meyer test. Values are expressed as mean ± SEM (Five mice per group). Comparisons—*** *p* < 0.001, and ns, non-significant vs. CRAB C0 control.

Based on its antibacterial, antibiofilm, and anti-inflammatory effects in LPS-RAW 264.7 cells, we further analyzed the efficacy of R-Pro10-1D as a potential antiseptic peptide in a mouse model of CRAB-induced septic shock. To address safety concerns, we initially analyzed the serum levels of toxicological markers such as aspartate aminotransferase (AST) and alanine aminotransferase (ALT) in the liver and blood urea nitrogen (BUN) in the kidneys of mice treated with R-Pro9-3D (1 and 5 mg/kg, 24 h). Neither dose of R-Pro9-3D elevated the levels of these enzymes (Figure 9A), confirming the non-toxic properties of the peptide even at a higher dose. Therefore, we conducted survival analyses using 5 mg/kg R-Pro9-3D and used 1 mg/kg to treat the mouse model of sepsis. Mice exposed to CRAB C0 (6 × 10^7^ CFU) displayed a 100% mortality rate 21 h after infection (Figure 9B); however, those treated with 5 mg/kg RPro9-3D displayed a 50% survival rate at 96 h.

In the sepsis model, mice infected with CRAB C0 (6 × 10^6^ CFU) had an increased bacterial load in vital organs such as the lungs, liver, and kidneys, which caused severe organ damage (Figure 9C). Moreover, CRAB C0 infection rapidly released excessive levels of endotoxins into the circulatory system (Figure 9D) and increased the serum levels of organ damage markers (AST, ALT, and BUN) and production of inflammatory cytokines (Figure 9E–K). Pretreatment with 1 mg/kg R-Pro9-3D significantly inhibited bacterial growth, as indicated by lower CFUs in the lysates of vital organs (Figure 9C). In addition, pretreatment with R-Pro9-3D reduced endotoxin levels by 30.5% in CRAB C0-infected mice (Figure 9D) and reduced AST, ALT, and BUN levels to 44.2%, 68.8%, and 78.6% compared to that in the bacterial control (Figure 9E–G). Next, we investigated the ability of R-Pro9-3D to regulate cytokine storms, observing significant down-regulation in TNF-α and IL-6 levels in serum and lung specimens compared to that in CRAB C0-infected mice (Figure 9H–K). R-Pro9-3D pretreatment also efficiently recovered pathological hallmarks induced by CRAB C0, such as significantly preventing neutrophil infiltration evidenced by lung microanatomical alterations (Figure 9L). Taken together, our findings suggest that R-Pro9-3D is a promising peptide antibiotic for treating carbapenem-resistant septic infections.

## 3. Discussion

The -prevalence of carbapenem-resistant Gram-negative bacteria is increasing, and CRAB poses a severe threat to global healthcare as it causes untreatable antibiotic-resistant infections [8]. Relatively few novel drugs or techniques are currently in development or in clinical trials for treating CRAB infections [12]. Therefore, new therapeutic approaches are required to halt the spread of antibiotic-resistant *A. baumannii* infections. AMPs have been proposed as potential replacements for conventional antibiotics when treating sepsis owing to their broad-spectrum bactericidal and immunomodulatory properties [15]. Unfortunately, the clinical application of AMPs is limited by their propensity for enzymatic degradation [51]; however, peptides with D-amino acid substitutions are completely resistant to proteolytic degradation in vivo, ensuring maximum bioavailability and therapeutic efficacy [52]. To achieve these properties, we previously developed Pro9-3D from the parent peptide Pro9-3, based on the insect defensin protaetiamycine, which displayed antibacterial efficacy but caused significant toxicity in mammalian cells [40,41]. Thus, simply substituting (L) for (D)-amino acids may be inefficient as it entirely alters sidechain orientations with respect to the target, preventing proper binding geometry and leading to detrimental consequences [53].

RI is a simple method for solving the proteolysis and toxicity issues associated with unstructured peptides by reversing the (D)-peptide sequence—flipping the termini and re-storing the (L)-amino side chain angles. This ensures that the peptide mimics the biological activity of the parent molecule while remaining proteolytically inert [54]. Using an RI approach, we designed R-Pro9-3 and R-Pro9-3D by reversing the parent sequence (Pro9-3D) and evaluated their specificity against Gram-negative bacteria, including CRAB strains. We found that R-Pro9-3D is an active peptide that exerts better antibacterial effects against CRAB strains, penetrates the cell membrane, binds firmly to LPS, exhibits good proteolytic stability with low cell cytotoxicity, targets macrophages, and induces anti-inflammatory effects and antiseptic immune responses in mice with CRAB C0-induced sepsis.

We postulate that R-Pro9-3 and R-Pro9-3D may eventually have better specificity toward Gram-negative bacterial strains, including carbapenem-resistant strains. As demonstrated in our study, R-Pro9-3D was a potent peptide that shared most of the features of Pro9-3D but appeared to have superior antibacterial effects, particularly against CRAB strains. Notably, R-Pro9-3D also showed a stronger activity than Pro9-3D and R-Pro9-3, suggesting that peptide sequence reversion and D-amino acid substitution contribute synergistically toward the antibacterial activity of R-Pro9-3D. Indeed, R-Pro9-3D showed outstanding potency (GM, 4.7) against 11 CRAB strains compared to Pro9-3D (GM, 7.6), whereas R-Pro9-3 (GM, 26.9) demonstrated significantly lower bacterial effects than Pro9-3 (GM, 25.6). Since the topology of the side chains of the RI analogue in the C-to-N orientation is the same as that of the parent peptide in the N-to-C orientation [55], our findings suggest that the greater antimicrobial activity of R-Pro9-3D compared to R-Pro9-3 may be mediated not only by the altered peptide side chains, but also by backbone orientation. Although the CD spectrum of R-Pro9-3D was an exact mirror image of its enantiomer, R-Pro9-3D had a slightly higher contents of α-helical structure in DPC micelles than Pro9-3D. Since peptide sequence reversion changes interactions between the sequential side chains, it may also alter peptide folding, causing the retro peptide, R-Pro9-3D, to have a more helical secondary structure than Pro9-3D. The high cationicity and amphipathic α-helical structure of R-Pro9-3D may explain its strong interaction with the amphipathic bacterial membrane, improving its antibacterial activity compared to Pro9-3D; however, further structural, and biophysical experiments are required to clarify this difference. 

The lytic activity of peptides against highly sensitive mammalian cells is an essential indicator of their toxicity and, therefore, their safety in clinical practice [56]. We found that R-Pro9-3D and the other analogs only generated around 5% hemolysis in red blood cells compared to melittin. R-Pro9-3D showed reduced cytotoxicity in mammalian cells and a higher relative selective index (31.7) than Pro9-3D, which showed significant cytotoxicity associated with a decrease in the relative selective index (25.0). Besides chirality, sidechains, and backbone orientation, RI may alter other properties related to bacterial cell selectivity and decreased cell cytotoxicity. Therefore, the intramolecular interactions as well as interactions with membrane need to be investigated to understand the characteristics of R-Pro9-3D in our future study [55].

We also investigated the antibacterial mechanism of the peptides using membrane permeability experiments. The ability of AMPs to engage with bacterial membranes or cell walls as a direct mechanism of cell death or as a method of reaching intracellular targets is crucial to their bactericidal and/or bacteriostatic activity [37,57]. LPS is a component of the outer membrane of Gram-negative bacteria that crucially affects pro-inflammatory activity by attaching to innate immune receptors (e.g., Toll-like receptors (TLRs)) [58]. Given the amphipathic and anionic nature of LPS, cationic peptides such as melittin and maganin have been shown to undergo significant electrostatic interactions with LPS [59]. As an amphipathic peptide, R-Pro9-3D can more effectively target LPS in the outer-membrane of Gram-negative bacteria via electrostatic interactions. Furthermore, membrane depolarization and FE-SEM analyses validated that R-Pro9-3D was able to translocate to the outer membrane and disrupt the membrane integrity of Gram-negative CRAB C0 more effectively. Thus, the RI peptide maintained its membrane insertion ability due to its amphiphilic nature. 

The primary causes of biofilm formation by MDR bacteria are antibiotic resistance and bacterial susceptibility to proteolytic cleavage. The efficacy of several AMPs as therapeutics is limited by their low structural stability and activity in physiological environments [60,61]. Here, we found that R-Pro9-3D remains totally intact under various digestive conditions and like Pro9-3D, exerts significant antibacterial activity against Gram-negative bacteria. This suggested that inversion and RI may have provided R-Pro9-3D with significant protection against proteolysis, as proteases are less likely to target peptide bonds containing D-amino acids [62]. Almost all ESKAPE pathogens, including *A. baumannii*, can form biofilms on biotic (e.g., skin, mucosa, and wounds) and abiotic (e.g., catheter) surfaces, resulting in drug resistance and persistent infections [45]. Previous studies have reported that AMPs namely LL-37, and more recently RI-analogue of Aurein 2.2 can possess biofilm prevention and degradation capacities [63,64,65]. Consistently, we found that R-Pro9-3D exerted more effective antibiofilm activity against CRAB C0 than Pro9-3D and had improved proteolytic stability. Thus, we suggest that the cell-permeable ability of R-Pro9-3D due to its amphipathic cationic properties may either retained or enhanced the antibiofilm activity [51]. Antibiotic resistance has been linked to a complex collection of factors, including bacterial outer membrane thickening, which limits antibiotic permeability, porin loss, antibiotic target site mutations, and efflux pump overexpression [8]. Notably, CRAB develops comparable mechanisms such as altered or lost outer membrane receptors, efflux pumps, and OXA-23 gene changes, all of which impact the use of currently available antibiotics against CRAB [14,66]. Combination therapy has lately introduced a strategy to treat infections caused by CRAB, making it easier to utilize and enhance the uptake of such resistance drugs [67,68]. It has been reported that a dual combination of colistin and imipenem exhibited the strongest synergistic efficacy by modulating the bacterial outer membrane [69]. Therefore, in our future study, we will examine the synergistic effect of R-Pro9-3D in combination with carbapenems to reduce the dose required for individual drugs, lowering the risk of drug toxicity.

Local cytokine production is coordinated in response to LPS assault, resulting in downstream TLR4 signaling activation and LPS-mediated endotoxemia [50]. Since R-Pro9-3D displayed a significant LPS-neutralizing capacity, it also inhibited the production of nitrite, TNF-α, and IL-6 in LPS-induced RAW 264.7 cells. Hence, R-Pro9-3D may largely neutralize LPS, rendering it inaccessible to the TLR4/MD-2 complex, and thereby regulate TLR4 activation. Infections with *A. baumannii*, an opportunistic Gram-negative bacterium, are common in immunocompromised patients, particularly those in intensive care units or undergoing invasive surgery. Antibiotic resistance (e.g., carbapenem antibiotics) has allowed *A. baumannii* to live in a hostile environment, resulting in its impact as a nosocomial pathogen [70]. Therefore, we evaluated the antiseptic effects of R-Pro9-3D using a mouse model of CRAB C0-induced sepsis, since CRAB can cause untreatable infections and pose a serious threat to human health. Notably, we found that R-Pro9-3D showed good efficacy by effectively reducing CRAB C0 growth in vivo and inhibiting the production of proinflammatory cytokines (TNF-α and IL-6) in the blood and lung lysates of infected mice. Furthermore, this study demonstrated for the first time that R-Pro9-3D protects infected mouse lung tissues from excessive neutrophil infiltration, confirming that R-Pro9-3D markedly reduces lung inflammation caused by CRAB C0 infection. Therefore, R-Pro9-3D displays robust antibacterial and anti-inflammatory effects in vitro and in vivo, as well as great bacterial cell selectivity. To have therapeutic potential in the treatment of sepsis, peptide post-treatment must be effective. In our future study, we need to investigate the efficacy of R-Pro9-3D in the post-treatment sepsis model as well as its potency against CRAB isolates from countries other than South Korea.

## 4. Materials and Methods 

### 4.1. Peptide Synthesis

All peptides were synthesized by solid-phase synthesis methodology using N-(9-fluorenyl) methoxycarbonyl amino acids and were purified by reversed-phase preparative high-performance liquid chromatography to >95%, characterized by matrix-assisted laser-desorption ionization-time-of-flight (MALDI-TOF) mass spectrometry at the Korea Basic Science Institute (KBSI, Ochang, Korea).

### 4.2. Bacterial Strains

*Escherichia coli* KCTC 1682 was obtained from the Korean Collection for Type Cultures (KCTC) (Jeongeup-si, Korea), *Acinetobacter baumannii* KCCM 40203, *Pseudomonas aeruginosa* KCCM 11328 from the Korea Culture Center of Microorganisms (KCCM) (Seoul, Korea). *Klebsiella pneumoniae* NCCP 16054 were obtained from the National Culture Collection for Pathogens (NCCP) (Osong, Korea). Additionally, two carbapenem-resistant *E. coli* (CREC E1 and E2), 11 carbapenem-resistant *A. baumannii* (CRAB C0–C10) and two carbapenem-resistant *K. pneumoniae* (CRKP K1 and K2) were isolated from the patients, who presented symptoms and signs of infection at Korea University Anam Hospital (Seoul, Korea) (IRB registration no. 2020AN0157, 4 March 2021). CRAB C0–C10 have the OXA-23 gene with carbapenem-resistance.

### 4.3. Measurement of Antibacterial Activity

The minimum inhibitory concentrations (MIC) were determined using broth dilution method. Briefly, all the mentioned bacterial strains were grown overnight at 37 °C by Muller–Hinton (MH) media. Peptides, melittin control and conventional antibiotics (Imipenem and meropenem) were added to the bacterial suspensions (2 × 10^5^ CFU/mL, MH media). After 16 h incubation, the bacterial growth was read at 600 nm using a SpectraMAX microplate reader (Molecular Devices, San Jose, CA, USA).

### 4.4. Protease Stability Assay

All the peptides at their MICs were pre-incubated with proteolytic enzymes such as trypsin and α-chymotrypsin (10,000:1; peptide: enzyme ratio) for 6 h at 37 °C. Then, 100 μL of peptide-protease cocktail was added to the 100 µL bacterial suspension (*E. coli*, *A. baumannii* and CRAB C0, 2 × 10^5^ CFU/mL) and further incubated for 16 h at 37 °C. Bacterial suspension without peptide and protease served as negative control. The analysis was repeated at three times using independent experiments, and the degree of bacterial growth was measured at 600 nm using a microplate reader.

### 4.5. Time-Killing Assay

The time-dependent bactericidal activities of Pro9-3, Pro9-3D, R-Pro9-3, R-Pro9-3D and melittin against *E. coli* and CRAB C0 strains. In brief, all the peptides were treated (MIC of R-Pro9-3D) to 2 × 10^5^ CFU/mL bacterial suspensions in MH medium at 37 °C. The time required to kill *E. coli* and CRAB C0 were monitored for up to 8 h. The survival rate of all the peptides treated bacterial supernatants were plated on a Luria–Bertani (LB) agar plate for 12 h at 37°C and expressed as percent bacterial killing.

### 4.6. Peptide–LPS Binding Assay 

The binding abilities of the peptides with LPS was analyzed by BODIPY-TR cadaverine (BC, Thermo Fisher Scientific, Waltham, MA, USA)-displacement assay [71]. The probe complex was prepared by mix 5 μg/mL BC with 5 μg/mL LPS (*E. coli* O111:B4, Sigma-Aldrich, St. Louis, MO, USA) in a 50 mM Tris buffer (pH 7.4) at room temperature for 6 h. Peptides including polymyxin B (PMB) control (1–64 μM) were allowed to interact with LPS–BC mix in a black 96-well plate for 30 min. The relative fluorescence intensity was recorded at an excitation wavelength of 580 nm and emission wavelength of 620 nm using a fluorescence microplate reader (Molecular Devices, San Jose, CA, USA).

### 4.7. Limulus Amebocyte Lysate (LAL) Assay

Peptide-mediated LPS neutralization was determined using LAL assay (GenScript, Piscataway, NJ, USA). Briefly, peptides 10 μL each (3.1, 6.3, 12.5, 25, and 50 μM) were allowed to interact with LPS (2 ng/mL) for 10 min at 37 °C. LL-37 was used as a control. A LAL enzyme (10 μL) was added to peptide-LPS complex for 10 min followed by addition of a chromogenic substrate (5 min, 37 °C). The reaction was stopped using color substrates and the absorbance was measured at 545 nm against endotoxin standard. The endotoxin levels are expressed as endotoxin units (EU) per milliliter.

### 4.8. CRAB Depolarization Assay

The depolarization capacity by peptides were measured by using a membrane potential sensitive dye 3,3′-dipropylthiadicarbocyanine iodide (diSC_3_-5) as previously described [72] using intact CRAB C0. Briefly, CRAB C0 cells were washed using wash buffer (5 mM HEPES, 20 mM glucose, pH 7.4). The cells were resuspended in dilution buffer (5 mM HEPES, 20 mM glucose, 0.1 M KCl, pH 7.4) and then incubated with diSC_3_-5 dye (1 h). In parallel, spheroplasts of CRAB C0 cells (contains plasma membrane and peptidoglycans) were prepared by damaging outer membrane using osmotic shock, as described previously [53]. Finally, varying concentrations of peptide treated cells, negative control (cells with dye) and positive control (1% triton X-100) were recorded for change in fluorescence using fluorescent spectrophotometer and expressed as percent depolarization.

### 4.9. Bacterial Outer Membrane Permeability Assay 

The effect of peptides on the disturbance of CRAB C0 outer membrane was analyzed using 1-N-phenylnaphthylamine (NPN, Sigma-Aldrich, St. Louis, MO, USA) uptake assay, as previously described [53]. Briefly, CRAB C0 cell suspensions were mixed with NPN (1 mM) and the background fluorescence was recorded for subtraction (excitation λ = 350 nm, emission λ = 420 nm) using RF-6000PC fluorescent spectrophotometer (Shimadzu Scientific Instruments, Kyoto, Japan). Changes in the NPN fluorescence were recorded after addition of different concentrations of peptides and values are expressed as—fluorescence intensity (A.U.).

### 4.10. Biofilm Assay

The effects of peptides on biofilm inhibition of *A. baumannii* and CRAB C0 strains were performed as described previously [73]. Briefly, bacterial cells (2 × 10^5^ CFU/mL in MH media containing 0.2% (*w*/*v*) glucose) were exposed with varying concentrations (0–64 μM) of peptides, melittin, imipenem and meropenem for 16 h at 37 °C. After treatment, bacterial cells were fixed with methanol (100%, 15 min) followed by crystal violet staining (0.1% (*w*/*v*) in 0.25% (*v*/*v*) acetic acid for 1 h). The plates were washed with distilled H_2_O, allowed to dry, and dissolved in ethanol (100% *v*/*v*). The color development representing the degree of biofilm was measured at 595 nm.

### 4.11. Circular Dichroism (CD) Analysis

All CD experiments for peptides were performed using a J-810 spectropolarimeter (Jasco, Tokyo, Japan) with a 1 mm path length cell at 25 °C. The CD spectra of the peptides at 100 µM were recorded in 0.1 nm intervals from 190 to 250 nm. The CD experiments were performed in aqueous solution and in 50 mM DPC micelles to investigate the structural changes as described previously [72]. Data from 10 scans were averaged for each CD spectrum and smoothed using J-810. CD data were expressed as the mean residue ellipticity (θ) in deg·cm^2^·dmol^−1^.

### 4.12. Hemolytic Aassay

The peptide-induced toxicity was determined by hemolytic against Sheep red blood cells (sRBC). Briefly, Fresh sRBC were washed at least three times with phosphate-buffered saline (PBS) and the debris were removed by centrifugation (1000× *g* for 5 min, 4 °C). All the peptides including melittin control (0.2–100 μM) and positive control (1% (*v*/*v*) triton X-100) were incubated with 4% (*v*/*v*) sRBC for 1 h at 37 °C followed by centrifugation at 1000× *g* for 5 min. The supernatant was measured at 405 nm using a microplate reader.

### 4.13. Cytotoxicity Assessment In Vitro

RAW 264.7 mouse macrophage cells and Human kidney (HK)-2 cells were obtained from Korean cell line bank (Seoul, Korea) and the cells were maintained in DMEM culture media (Thermo Fischer Scientific Inc., Waltham, MA, USA) supplemented with 10% fetal bovine serum, 1% penicillin/streptomycin at 37 °C in a humidified 5% CO_2_ incubator. The cytotoxicity of the peptides was analyzed using WST-8 cell proliferation assay (Biomax Co, Ltd., Seoul, Korea), and the experiment was performed according to the kit protocol. Briefly, cells (1 × 10^4^) were seeded in 96-well plate and the peptides treatment (0–100 μM) were initiated at 80% confluency and then for 24 h. After incubation, WST-8 reagent was added and the change in absorbance was read at 450 nm against reagent blank, and values are expressed as percent cell survival.

### 4.14. Scanning Electron Microscope Analysis

Ultrastructural examination of CRAB C0 membrane disturbance by R-Pro9-3D was visualized by field emission-scanning electron microscopy (FE-SEM) as previously described [74]. Briefly, CRAB C0 at mid-log phase (OD_600_ of 0.2) were incubated with 4 μM and 8 μM of R-Pro9-3D in MH media for 30 min or 1 h at 37 °C. The cells were washed and fixed in 2.5% (*v*/*v*) glutaraldehyde. After overnight fixation, cells were PBS washed and again fixed in 1% (*v*/*v*) osmium tetroxide for 1 h. After series of dehydration process, cells were finally coated with platinum and observed for topological changes under FE-SEM (SU8020; Hitachi, Tokyo, Japan).

### 4.15. Quantification of Nitrite and Inflammatory Cytokine Production in LPS-Stimulated RAW264.7 Cells

The effect of peptides on inhibition of nitrite production was assessed by Griess assay. Briefly, RAW264.7 cells (1 × 10^5^) were pre-treated with varying concentration of peptides (1, 5, 25, and 50 µM) for 1 h and then stimulated with 20 ng/mL of LPS for 16 h. After incubation, an equal ratio of supernatant and Griess reagent was added. The change in color formation was read at 540 nm. The concentration of nitrite content was assessed using a standard curve of sodium nitrite. In parallel, release of inflammatory cytokines including TNF-α and IL-6 in the culture media was quantified using an enzyme-linked immunosorbent assays kit (ELISA; R&D Systems, Minneapolis, MN, USA) and the assay was performed according to the kit protocol. All the analyses were conducted in triplicate, and the concentrations of TNF-α and IL-6 were evaluated by measuring the absorbance at 450 nm using a microplate reader.

### 4.16. Animals

Female ICR mice were purchased from Orient (Daejeon, Korea) and were housed under specific pathogen-free and humidity-controlled environment. All procedures were reviewed and approved by the Institutional Animal Care and Use Committee (IACUC) of Konkuk University, South Korea (IACUC number: KU20192; 2021.04.05).

### 4.17. In Vivo Toxicity Measurements

The ICR mice (*n* = 5 per group) were intraperitoneally (i.p.) injected with R-Pro9-3D (1 mg and 5 mg/kg/day in PBS) and the serum levels of AST, ALT, and BUN levels were determined using a standard kit from Asan Pharmaceutical as described previously [74].

### 4.18. Survival Analysis

Forty ICR mice were sorted into four groups (10 mice per group). Mice intraperitoneally (i.p.) injected with PBS served as normal control. Mice received 10-fold increment of CRAB C0 (6 × 10^7^ CFU/mouse) enrolled as bacterial control, peptide control-R-Pro9-3D (5 mg/kg). Treated mice received R-Pro9-3D 1 h before CRAB C0 injection and the survival rate of all experimental mice were examined at 3 h intervals for 96 h.

### 4.19. CRABC0 Sepsis Mouse Model

Twenty ICR mice were randomly divided in to 4 groups (5 mice per group). PBS alone mice served as normal control. Peptide control mice received i.p. injections of R-Pro0-3D (1 mg/kg). Mice received only CRAB C0 (6 × 10^6^ CFU/mice) act as bacterial control. For peptide treatment groups, R-Pro9-3D was injected 1 h before CRAB C0 injection. After 16 h of treatment, mice were killed by euthanasia and the lungs, liver, and kidneys were removed aseptically and then homogenized using ice-cold PBS. To assess the relative abundance of CRAB C0, all homogenates (1:1000, PBS) were plated onto Luria–Bertani agar, and the numbers of bacteria colonies were counted [42]. The levels of inflammatory cytokines (TNF-α and IL-6) were measured in the serum and lung lysates using corresponding ELISA kits (R&D Systems, Minneapolis, MN, USA). The contents of AST, ALT, and BUN were determined using a standard kit from Asan Pharmaceutical, as described previously [74]. To determine microanatomical features of polymorpho-neutrophil infiltrations, 5 μm-thick sections were prepared from paraffin blocked lungs and sequentially processed for hematoxylin and eosin (H&E) staining and examined using light microscope.

### 4.20. Statistical Analysis 

All the experiments were performed at least three times using independent experiments (mean ± standard errors of the mean (SEM)). Data were analyzed by Kaplan–Meier log-rank test, one-way ANOVA and two-way ANOVA followed by Dunnett’s tests using GraphPad Prism (GraphPad Software Inc., La Jolla, CA, USA). Values indicate statistically significant at * *p* < 0.05, ** *p* < 0.01, and *** *p* < 0.001; ns represents non-significant.

## 5. Conclusions

In this study, we developed potent short 9-meric peptides with high selectivity and low cytotoxicity for treating CRAB infections. R-Pro9-3D designed by a retro inversion strategy showed rapid and effective antibacterial activity against Gram-negative bacteria, particularly clinical CRAB isolates, by permeabilizing bacterial membrane. Furthermore, R-Pro9-3D demonstrated good proteolytic stability, low cytotoxicity, and in vivo protective activity against lethal CRAB C0 infection. Thus, we believe that R-Pro9-3D has the potential to be an effective antiseptic therapeutic peptide for treating CRAB infections and this study creates the basis for the development of novel antimicrobial drugs.

## Figures and Tables

**Table 1:** Peptides and their physicochemical properties.

Peptides	Sequence ^a^	Length	Molecular Weight	Charge	Hydrophobic Moment <µH> ^b^	Hydrophobicity <H> ^b^
Pro9-3	RLWLAIWRR-NH2	9	1269	+3	0.692	0.776
Pro9-3D	rlwlaiwrr-NH2	9	1269	+3	0.692	0.776
R-Pro9-3	RRWIALWLR-NH2	9	1269	+3	0.692	0.776
R-Pro9-3D	rrwialwlr-NH2	9	1269	+3	0.692	0.776

^a^ Small letters in sequence represents D-amino acids. ^b^ Hydrophobic moment <µH> and hydrophobicity <H> were calculated by http://heliquest.ipmc.cnrs.fr/cgi-bin/ComputParams.py; Date accessed: 12 September 2021.

**Table 2:** Antibacterial activities of R-Pro9-3 peptides in comparison with those of Pro9-3 peptides against of standard Gram-negative bacteria and carbapenem-resistant Gram-negative bacteria.

Microorganisms	Minimal Inhibitory Concentration (MIC) in μM						
	Pro9-3	Pro9-3D	R-Pro9-3	R-Pro9-3D	Melittin	Imipenem	Meropenem
Standard Gram-negative bacteria							
*E. coli*	16	8	16	8	8	0.5	0.25
*A. baumannii*	16	4	16	4	4	1	0.5
*P. aeruginosa*	64	8	64	8	16	1	1
*K. pneumoniae*	64	8	32	8	32	<0.25	<0.25
Carbapenem-resistant Gram-negative bacteria							
CREC E1	16	8	16	8	8	>128	>128
CREC E2	16	8	16	8	8	16	16
CRAB C0	8	4	8	4	4	>128	128
CRAB C1	16	8	32	8	4	>128	>128
CRAB C2	16	8	32	4	4	>128	128
CRAB C3	16	8	32	8	4	128	128
CRAB C4	16	8	32	4	4	128	>128
CRAB C5	16	8	32	4	4	128	>128
CRAB C6	16	8	32	4	4	128	>128
CRAB C7	16	8	32	4	4	128	128
CRAB C8	16	8	32	4	4	>128	>128
CRAB C9	16	8	16	4	4	>128	>128
CRAB C10	16	8	16	4	4	>128	>128
CRKP K1	64	16	32	16	64	32	64
CRKP K2	64	8	32	8	64	>128	>128
^a^ GM	25.6	8.0	27.4	6.3	13.1	90.3	91.9
^b^ HC_10_	200	200	200	200	3.1	na	na
^c^ Relative selective index	7.8	25.0	7.3	31.7	0.2	na	na

^a^ The geometric means (GM) are the mean minimum inhibitory concentrations (MICs) values of all bacterial strains. ^b^ HC_10_ is the degree of peptide concentration inducing 10% hemolysis of heparinized sheep red blood cells in vitro. ^c^ Relative selective index is the minimal peptide concentration that produces 10 percent hemolysis. When no detectable hemolysis was observed at 100 μM, a value of 200 μM was used to calculate the selective index and was calculated using HC_10_/GM of the MIC (μM). The larger values indicate greater cell selectivity and na, not applicable. CREC, Carbapenem-resistant *E. coli*; CRAB, Carbapenem-resistant *A. baumannii*; CRKP, Carbapenem-resistant *K. pneumoniae*.

**Table 3:** Inhibition of antimicrobial activity of Pro9-3 and its analogs by trypsin and α-chymotrypsin against *E. coli*, *A. baumannii* and CRAB C0.

Microorganisms	Minimal Inhibitory Concentration (MIC) in μM			
	Pro9-3	Pro9-3D	R-Pro9-3	R-Pro9-3D
*E. coli* + Trypsin	>64	8	>64	8
*E. coli* + α-Chymotrypsin	>64	8	64	8
*A. baumannii* + Trypsin	>64	4	>64	4
*A. baumannii* + α-Chymotrypsin	>64	4	>64	4
CRAB C0 *+* Trypsin	>64	4	64	4
CRAB C0 *+* α-Chymotrypsin	>64	4	64	4

## Data Availability

The data presented in this study are available on request from the corresponding author.
